# Detecting amyloidosis at its dawn: novel quantum protein probes unveiled

**DOI:** 10.1097/MS9.0000000000004339

**Published:** 2025-11-14

**Authors:** Muhammad Talha, Maliha Khalid, Aminath Waafira

**Affiliations:** a Department of Medicine, King Edward Medical University, Lahore, Pakistan; b Department of Medicine, Jinnah Sindh Medical University,Karachi, Pakistan; c Department of Medicine, The Maldives National University, Malé, Maldives

*To the Editor*,

Amyloidosis that results in pathological fibrous aggregation due to misfolded proteins forms the foundation for several neurodegenerative disorders, especially Alzheimer’s disease. Amyloid accumulates silently and, over the years, manifests itself as clinical symptoms. Early diagnosis is crucial for intervention to improve outcomes. The current positron emission tomography (PET)-imaging and cerebrospinal fluid biomarker methods typically recognize amyloid pathology following gross neurodegeneration and limit their usage for identification in asymptomatic phases^[1]^. This warrants the need to build sensitive and specific tools for the early detection of such a disease-like condition.

Quantum protein probes are nanoscale agents that employ quantum mechanical properties for biomolecular detection. They are specifically quantum dot (QD)-based probes, which are highly popular and progressive in the aspect of a tool for early amyloid detection. QDs are semiconductor nanocrystals with exclusive optical properties, namely size-tunable fluorescent emission and exceptional photostability, allowing highly sensitive detection of amyloid aggregates. Unlike conventional amyloid dyes, such as thioflavin T, QD-based probes have the advantage of selective binding to neurotoxic species, namely, amyloid-beta (Aβ) monomers and oligomers, before fibril formation, thus providing insight into the pre-symptomatic disease state^[2,3]^. The process forms a binding event involving surface chemistry, hydrophobic interaction, and structure-specific recognition, followed by an increase in selectivity against amyloid species. Furthermore, differing amyloid strain types show distinctive fluorescence signatures and, subsequently, this could help determine an accurate diagnosis in a variety of amyloidopathies, as different strains may associate with different trajectories of disease progression^[3,4]^.

**HIGHLIGHTS**

- Quantum dot-based protein probes offer high sensitivity and specificity for early detection of amyloid aggregates, especially Aβ monomers and oligomers, before clinical symptoms appear.
- Real-time, *in vitro* and *in vivo* monitoring of amyloidogenesis is possible through fluorescence changes, aiding early diagnosis and treatment response tracking.
- Integration with current biomarker platforms and overcoming translational challenges could pave the way for personalized, proactive management of amyloid-related neurodegenerative diseases.

Mechanistically, QD probes bind to amyloidogenic proteins and cause fluorescence changes that correlate with amyloid conformation and concentration, thus allowing real-time, quantitative monitoring of amyloidogenesis both *in vitro* and *in vivo*. For example, graphene QDs have been employed in the reliable monitoring of kinetics for Aβ aggregation, which provides important insight into the progression of the disease and therapeutic response^[5]^. Recent advances in the detection of amyloid in biological fluids have been made with sensitivities potentially relevant for early biomarkers, thanks to a unique dual-probe fluorescence with QD conjugates technique^[6]^.

Although these probes are promising, the clinical translation does face challenges such as achieving biocompatibility, efficient blood-brain barrier penetration, and standardization of imaging protocols. The ongoing preclinical research is encouraging, yet the small sample size and variability of the experimental models are limitations that may detrimentally affect the broader applicability of the results. Current studies are, for example, directed toward surface modifications such as applying polyethylene glycol coatings for QD biocompatibility and BBB penetrability^[7]^. Interdisciplinary collaboration should be implemented to facilitate probe design optimization, the establishment of protocols, and the validation of diagnostic accuracy in large clinical studies. QD probes in conjunction with neuroimaging and CSF biomarker platforms would therefore benefit greatly from a QD-based fluorescence spectroscopy approach to amyloid PET imaging, although such full-fledged workflows are currently under development. Combining quantum protein probes with the existing neuroimaging and fluid biomarker platforms will become the basis for transforming early diagnosis and monitoring of amyloidosis toward personalized intervention^[8]^.

To summarize, quantum protein probes appear to afford pre-symptomatic detection of amyloidosis with greater sensitivity and specificity. Future work should include hybrid imaging and multi-analyte probes for improving accuracy. Medical research industry stakeholders and funding agencies will need to facilitate the translational studies for probe development and clinical validation. This could engender a paradigm shift for neurodegenerative disorders from reaction toward prevention. Our research aligns with the TITAN Guidelines toward transparency regarding artificial intelligence use in healthcare^[9]^.

## Data Availability

No data were generated for this manuscript.

## Research registration unique identifying number (UIN)

1. Name of the registry: Not applicable. 2. Unique Identifying number or registration ID: Not applicable. 3. Hyperlink to your specific registration (must be publicly accessible and will be checked): Not applicable.
